# Predictors of PASI90 response in patients with psoriasis treated with ınterleukin ınhibitors: observational cohort study^☆^

**DOI:** 10.1016/j.abd.2025.501132

**Published:** 2025-06-18

**Authors:** Burhan Engin, Yusuf Demir, Sera Nur Yucesoy, Tumay Ak

**Affiliations:** a Department of Dermatology, Istanbul University-Cerrahpasa, Cerrahpasa Medical Faculty, Istanbul, Turkey; b Department of Internal Medicine, Istanbul University-Cerrahpasa, Cerrahpasa Medical Faculty, Istanbul, Turkey

**Keywords:** Biological therapies, Psoriasis, Proportional hazards models, Treatment outcome

## Abstract

**Background:**

Psoriasis is a chronic multisystem disorder, and the inhibition of different cytokine pathways has been associated with different treatment responses.

**Objective:**

To demonstrate independent predictors of PASI90 response in patients with psoriasis under biologic therapy and compare the effectiveness of different biologic classes

**Methods:**

This cross-sectional study was conducted in a single tertiary center between January 2023 and May 2024 and included 623 patients (M/F: 320/303). PASI90 response was the primary end-point of the study, and PASI100 was the secondary end-point. Univariate and multivariate cox-regression analyses were used to identify predictors of PASI90. The efficacy of different biologic classes for achieving PASI90 and PASI100 responses was assessed using the Kaplan–Meier method.

**Results:**

The age of disease onset (HR = 1.037, 95% CI [1.029‒1.044], p < 0.001) and being biologic-naïve (HR = 1.261, 95% CI [1.046‒1.521], p = 0.015) were identified as significant predictors of PASI90 response. IL23 inhibitors showed considerably superior efficacy in achieving PASI90 response than both TNF inhibitors (p = 0.042) and ustekinumab (p = 0.027). Also, IL17 inhibitors exhibited near-significantly higher effectiveness than TNF inhibitors (p = 0.090) and ustekinumab (p = 0.050). The performance of biologic classes was not substantially different in reaching PASI100 overall.

**Study limitations:**

The most important limitation of this study is the retrospective data collection.

**Conclusion:**

Age of disease onset and being biologically naïve were positively associated with achieving PASI90 response, whereas ustekinumab use was negatively associated. Age of disease onset was the strongest predictor of PASI90. Also, IL23 and IL17 inhibition, especially IL23, appeared to provide a better therapeutic response.

## Introduction

Psoriasis is a chronic inflammatory disease that may affect the skin and musculoskeletal system. Skin psoriasis is characterized by the presence of erythematous plaque lesions, which tend to affect the extensor surfaces of the extremities. In addition, pustular and erythrodermic morphologies represent rarer involvement types.^1^ Special areas of involvement include the face, scalp, genitalia, palms, soles, and nails, which might be challenging to treat and substantially lower health-related quality of life.^2^ The complex interplay of cytokines resulting in rapid turnover of keratinocytes has been implicated in the pathogenesis of skin psoriasis.^3^ On the other hand, this inflammatory cascade can lead to systemic inflammatory signs and arthritis as well. Tumor Necrosis Factor (TNF), Interleukin (IL)17, and IL23 are major inflammatory cytokines acting as therapeutic targets for biological agents, and their inhibition provides favorable treatment responses.^4^

The PASI (Psoriasis Area and Severity Index) is a physician-assessed tool to measure the extent and severity of skin involvement in psoriasis.^5^ It is also a widely used scale to evaluate treatment response. The PASI score ranges from 0 to 72, with higher scores indicating more severe disease.^5^ In clinical practice, achieving PASI75 is often considered a meaningful treatment response.^6^ With the introduction of biologic agents in the treatment of psoriasis, the so-called biologic era, treatment expectations of physicians and patients increased, and achieving PASI90 response has become the new treatment target.^7^ PASI90 represents a 90% reduction in the PASI score from baseline and confers near-total clearance of psoriatic lesions. Furthermore, Randomized Controlled Trials (RCTs) evaluating the efficacy of biological treatments have increasingly started to assess effectiveness based on achieving a minimum PASI90 response.^8^, ^9^

TNF inhibitors have long been used to treat psoriasis with satisfactory PASI75 responses.^10^ Subsequent biologic agents targeting the different cytokine pathways expanded the therapeutic armamentarium of psoriasis, as different biologics provided better healing of distinct aspects of the disease. However, knowledge of whether there are differences in the efficacies of these agents and how the authors can predict PASI90 response is currently limited. In this study, the objective was to investigate predictive factors influencing the achievement of PASI90 response with biological treatments in psoriasis. Also, the authors compared the efficacy of different classes of biologics to reach PASI90 and PASI100 responses, so the authors aimed to see whether there are any differences regarding treatment effectiveness between pathogenesis-directed therapies.

## Materials and methods

This cross-sectional study was conducted between January 2023 and June 2024 in a tertiary center in Turkey (Cerrahpasa Medical Faculty Psoriasis Diagnosis and Treatment Unit). Psoriasis patients using a biologic agent who were more than 18 years of age and consecutively seen in the psoriasis outpatient clinic were included in the study. Patients were diagnosed with psoriasis according to their clinical manifestations by an experienced dermatologist BE; then, a histopathological confirmation was made. Biologic-naïve or biologic-experienced patients were included in the study. Biologic-experienced patients consisted of patients who used at least one biologic agent prior to the latest biologic treatment and switched to another biologic agent due to insufficient response or adverse effects. Biologic agents were administered per the standard dosing scheme in Table 1.

**Table 1:** Administration of biologic agents.

Medication	Dosing schedule
TNF inhibitors	
Adalimumab	80 mg SC at week-0, 40 mg at week-1, then 40 mg SC q2w
Infliximab	5 mg/kg IV at weeks 0, 2 and 6 and thereafter every 8-weeks
Certolizumab Pegol	Patient weight >90 kg: 400 mg SC q2w
Etanercept	50 mg SC twice weekly for first 12-weeks, then 50 mg SC weekly
IL-17 inhibitors	
Secukinumab	300 mg SC at weeks 0, 1, 2, 3, 4, then 300 mg SC q4w
Ixekizumab	160 mg SC at week-0, 80 mg at week 2, 4, 6, 8, 10, 12, then 80 mg SC q4w
IL-23 inhibitors	
Guselkumab	100 mg SC at week 0 and 4, then 100 mg q8w
Risankizumab	150 mg SC at week 0 and 4, then 150 mg q12w
IL-12/23 inhibitor	
Ustekinumab	Patient weight <100 kg: 45 mg SC at week 0 and 4, then 45 mg SC q12w
	Patient weight >100 kg, 90 mg SC at week 0 and 4, then 90 mg SC q12w

TNF, Tumor Necrosis Factor; IL, Interleukin; SC, Subcutaneous; IV, Intravenous.

Treatment responses with the actively used biologic agent (the last biologic therapy) were evaluated by BE, YD, and SNY, and time to reach PASI90 and PASI100 were recorded. Achieving the PASI90 response was the primary end-point of this study, and PASI100 was the secondary end-point. Clinical characteristics of patients were retrospectively collected from patients' charts. Patients administering concomitant oral immunomodulatory drugs such as methotrexate or leflunomide, having PASI scores less than five at the beginning of the last biologic agent, and receiving the latest biologic treatment for less than one month were excluded from the study. This study was approved by the Ethics Committee of Istanbul University-Cerrahpaşa, Cerrahpaşa Medical Faculty, and was conducted following the ethical principles of the Declaration of Helsinki (ethics approval nº 840876)

### Statistical analysis

All analyses were performed using IBM SPSS Statistics for Windows, Version 23.0 (IBM Corp., Armonk, NY, USA). Normality analyses of the data were performed using the Kolmogorov-Smirnov test, Shapiro-Wilks test, and visual histograms. Categorical variables were shown as numbers and percentages. While normally distributed continuous variables were shown as mean ± Standard Deviation (SD), non-normal distributed continuous variables were given as median (min‒max). The performance of different biologic classes to achieve PASI90 and PASI100 was evaluated using Kaplan-Meier analysis with Log-rank test. Cox regression analysis was used to detect independent predictors of PASI90 response. A required sample size of the total number of events for the proportional hazards regression model was calculated as 429 at 0.05 alpha and 0.05 beta error rate.^11^ Since anti-TNFs are the first class approved to be used in psoriasis,^12^ this biologic class was accepted as a reference category for the regression model. Biologic classes and other covariates were initially evaluated with univariate analysis using the enter method. Then, covariates meeting the significance level of p < 0.25 were further analyzed with multivariate analysis using the forward stepwise method to constitute different prediction models; p < 0.05 was regarded as statistically significant.

## Results

Patient characteristics are summarized in Table 2. This study included 623 patients (M/F: 320/303), and their median age was 40 (min‒max: 18‒72). The median age of disease onset was 23 (min‒max: 8‒58), and the disease duration was 15 (min‒max: 0.1‒68) years. Plaque psoriasis was the most common psoriasis morphology (97%). Extremities (85%) were the most commonly involved area, followed by the trunk (73%) and scalp (55%), and 30% of patients had psoriatic arthritis. Half of the patients were biologic-naïve, and there were significant difference regarding the number of biologic-naïve patients across the biologic classes (anti-TNFs [n = 55, 82%], anti-IL17 [n = 105, 62%], anti-IL23 [n = 129, 39%], and anti-IL12/23 [n = 29, 53%], p < 0.001). Anti-IL23 agents (53%) were most frequently used, followed by anti-IL17 class (26%). Within the anti-IL23 class, rates of risankizumab (27%) and guselkumab (26%) were almost the same. Also, anti-IL17 agents, secukinumab (13%) and ixekizumab (13%), were used at the same rates. While anti-TNFs were used in 10% of patients, anti-IL12/23 in 9%. Adalimumab (7%) was the most commonly used agent in the anti-TNF class. The median duration of using the last biologic agent was 16 (min‒max: 1‒72) months. The most frequent comorbidity was obesity (18%), and this was followed by hypertension (12%) and diabetes mellitus (10%). PASI90 was achieved in 519 (83%) patients and PASI100 in 278 (45%).

**Table 2:** Patient characteristics.

Features		Total (n = 623)
Sex (M/F)		320/303
Age, median (min‒max)		40 (18–72)
BMI (kg/m^2^), median (min‒max)		25.5 (14.2–49.2)
Smoking, n (%)		295 (47)
Age of disease onset, median (min‒max)		23 (8–58)
Disease duration, years, median (min‒max)		15 (0.1–68)
Family history of psoriasis: n(%)		264 (42)
Psoriasis morphology	Plaque, n (%)	608 (97)
	Pustular, n (%)	11 (2)
	Erythrodermic, n (%)	4 (0.6)
Special areas of involvement	Genital, n (%)	151 (24)
	Scalp, n (%)	344 (55)
	Nail, n (%)	231 (37)
	Face, n (%)	232 (37)
	Palmoplantar, n (%)	91 (15)
Trunk involvement, n (%)		456 (73)
Extremity involvement, n (%)		530 (85)
Psoriatic arthritis, n (%)		190 (30)
Biologic-naïve, n (%)		318 (51)
PASI score just before the last biologic therapy, median (min‒max)		24 (5–70)
Last biological treatments of patients		
Anti-TNFs	Adalimumab, n (%)	46 (7)
	Etanercept, n (%)	6 (1)
	Certolizumab pegol, n (%)	8 (1)
	Infliximab, n (%)	7 (1)
Anti-IL17	Secukinumab, n (%)	86 (13)
	Ixekizumab, n (%)	84 (13)
Anti-IL23	Risankizumab, n (%)	169 (27)
	Guselkumab, n (%)	163(26)
Anti-IL12/23	Ustekinumab, n (%)	54 (9)
Duration of treatment with the last biologic agent, months, median (min‒max)		16 (1–72)
PASI 90 response, n (%)		519 (83)
PASI 100 response, n (%)		278 (45)
Comorbidities	Diabetes mellitus, n (%)	63 (10)
	Hypertension, n (%)	75 (12)
	Dyslipidemia, n (%)	41 (7)
	Obesity, n (%)	112 (18)
	Ischemic heart disease, n (%)	27 (4)
	Asthma/COPD, n (%)	26 (4)
	Thyroid dysfunction, n (%)	46 (7)

M, Male; F, Female; BMI, Body Mass Index; PASI, Psoriasis Area Severity Index; TNF, Tumor Necrosis Factor; IL, Interleukin; COPD, Chronic Obstructive Pulmonary Disease.

**Fig. 1:**
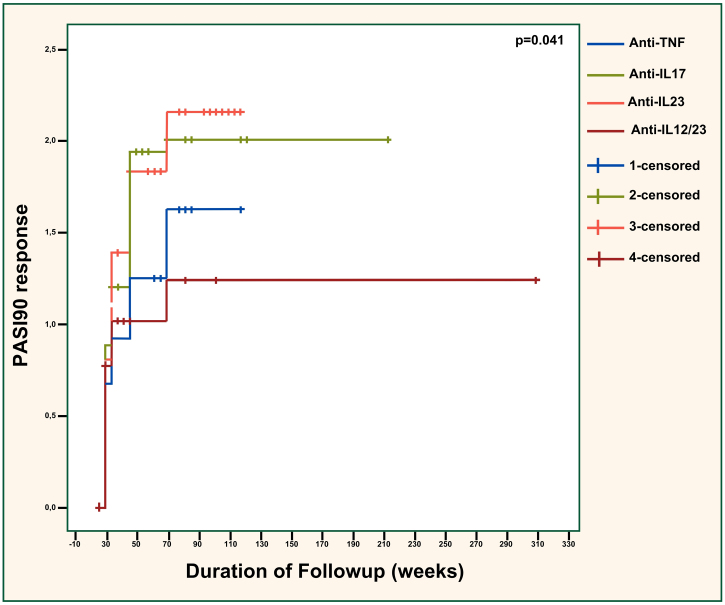
PASI90 response across different biologic classes. PASI, Psoriasis Area severity Sındex; TNF, Tumor Necrosis Factor; IL, Interleukin. Anti-TNF vs. anti-IL17; p = 0.090. Anti-TNF vs. anti-IL23; p = 0.042. Anti-TNF vs. anti-IL12/23; p = 0.656. Anti-IL17 vs. anti-IL23; p = 0.776. Anti-IL17 vs. anti-IL12/23; p = 0.050. Anti-IL23 vs. anti-IL12/23; p = 0.027.

**Fig. 2:**
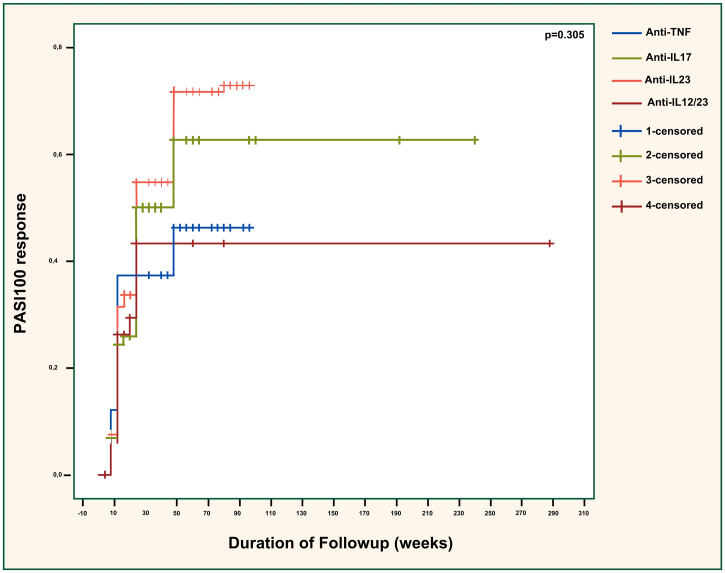
PASI100 response across different biologic classes. PASI, Psoriasis Area Severity Index; TNF, Tumor Necrosis Factor; IL, Interleukin. Anti-TNF vs. anti-IL17; p = 0.416. Anti-TNF vs. anti-IL23; p = 0.120. Anti-TNF vs. anti-IL12/23; p = 0.832. Anti-IL17 vs. anti-IL23; p = 0.320. Anti-IL17 vs. anti-IL12/23; p = 0.668. Anti-IL23 vs. anti-IL12/23; p = 0.294.

PASI90 responses were compared across the different biologic classes in Fig. 1 and showed significant differences (p = 0.041). The efficacy of anti-TNFs was significantly inferior to anti-IL23 agents (p = 0.042) but similar to anti-IL12/23 agents (p = 0.656). Also, anti-IL23 agents showed considerably superior efficacy than ustekinumab (p = 0.027). The effectiveness of anti-IL17 agents was almost significantly better than anti-TNFs (p = 0.090). Additionally, the difference between anti-IL17 and anti-IL12/23 agents was near-significant (p = 0.050), with anti-IL17 agents being superior. Anti-IL17 and anti-IL23 agents had similar effectiveness overall (p = 0.776). There was no significant difference regarding the PASI100 response between different biologic classes (p = 0.305), as demonstrated in Fig. 2.

In univariate analysis, age of disease onset (HR = 1.034, 95% CI [1.027‒1.041], p < 0.001), and palmoplantar involvement (HR = 1.034, 95% CI 1.027‒1.041), p = 0.042) were found to be substantially associated with a higher probability of achieving PASI90. On the other hand, extremity involvement (HR = 0.765, 95% CI [0.596‒0.980], p = 0.034) and ustekinumab use (HR = 0.611, 95% CI [0.394‒0.946], p = 0.027) were significantly related to a lower probability of achieving PASI90 response. Multivariate analysis yielded three different models for predicting PASI90 (Table 3). Model 3 identified three independent predictors for predicting PASI90 response in patients treated with biologic agents. While the age of disease onset (HR = 1.037, 95% CI [1.029‒1.044], p < 0.001) and being biologic-naïve (HR = 1.261, 95% CI [1.046‒1.521], p = 0.015) were positively associated with PASI90, using ustekinumab (HR = 0.560, 95% CI [0.358‒0.876], p = 0.011) was negatively associated. However, treatment with anti-IL17 (HR = 0.995, 95% CI [0.702‒1.410], p = 0.977) or anti-IL23 (HR = 1.238, 95% CI [0.895‒1.712], p = 0.197) agents did not significantly differ in terms of PASI90 response relative to anti-TNFs.

**Table 3:** Predictors for PASI90 response.

Covariates	Univariate analysis, HR (95% CI)	p	Multivariate analysis					
			Model 1, HR (95% CI)	p	Model 2, HR (95% CI)	p	Model 3, HR (95% CI)	p
Female gender	1.021 (0.859–1.214)	0.814	‒	‒	‒	‒	‒	‒
Age of disease onset	1.034 (1.027–1.041)	**<0.001**^c^	1.035 (1.028–1.043)	<0.001^c^	1.036 (1.029–1.044)	<0.001^c^	1.037 (1.029–1.044)	<0.001^c^
Smoking	1.032 (0.868–1.227)	0.719	‒	‒	‒	‒	‒	‒
BMI (kg/m^2^)	0.998 (0.979–1.018)	0.869	‒	‒	‒	‒	‒	‒
Pustular/erythrodermic morphology	0.965 (0.603–1.545)	0.884	‒	‒	‒	‒	‒	‒
Family history of psoriasis	0.971 (0.819–1.151)	0.733	‒	‒	‒	‒	‒	‒
PASI score just before the last biologic therapy	0.999 (0.992–1.006)	0.777	‒	‒	‒	‒	‒	‒
Special areas of involvement								
Genital	0.842 (0.689–1.029)	0.092	‒	‒	‒	‒	‒	‒
Scalp	0.996 (0.837–1.185)	0.964	‒		‒	‒	‒	‒
Nail	0.860 (0.719–1.029)	0.100	‒	‒	‒	‒	‒	‒
Face	0.834 (0.695–1.001)	0.051	‒	‒	‒	‒	‒	‒
Palmoplantar	1.293 (1.010–1.657)	**0.042**^a^	‒	‒	‒	‒	‒	‒
Trunk involvement	0.970 (0.797–1.181)	0.761	‒	‒	‒	‒	‒	‒
Extremity involvement	0.765 (0.596‒0.980)	**0.034**^a^	‒	‒	‒	‒	‒	‒
Psoriatic arhtritis	0.892 (0.740–1.076)	0.232	‒	‒	‒	‒	‒	‒
Biologic-naïve	1.118 (0.941–1.329)	0.205	‒	‒	‒	‒	1.261 (1.046–1.521)	**0.015**^a^
Class of biologic treatment								
Anti-TNF	Reference category							
Anti-IL17	0.996 (0.721–1.376)	0.983	‒	‒	0.935 (0.662–1.320)	0.703	0.995 (0.702–1.410)	0.977
Anti-IL23	1.173 (0.867–1.586)	0.300	‒	‒	1.112 (0.813–1.521)	0.505	1.238 (0.895–1.712)	0.197
Anti-IL12/23	0.611 (0.394‒0.946)	**0.027**^a^	‒	‒	0.534 (0.342‒0.834)	**0.006**^b^	0.560 (0.358‒0.876)	**0.011**^a^
Comorbidities			‒	‒	‒	‒	‒	‒
Diabetes mellitus	0.777 (0.578–1.044)	0.095	‒	‒	‒	‒	‒	‒
Hypertension	0.773 (0.584–1.022)	0.071	‒	‒	‒	‒	‒	‒
Dyslipidemia	0.901 (0.636–1.276)	0.556	‒	‒	‒	‒	‒	‒
Obesity	0.895 (0.716–1.118)	0.329	‒	‒	‒	‒	‒	‒
Coronary artery disease	0.803 (0.524–1.232)	0.315	‒	‒	‒	‒	‒	‒
Asthma/COPD	0.850 (0.554–1.303)	0.455	‒	‒	‒	‒	‒	‒
Thyroid dysfunction	0.798 (0.571–1.116)	0.188	‒	‒	‒	‒	‒	‒

PASI, Psoriasis Area Severity Index; HR, Hazard Ratio; CI, Confidence Interval; BMI, Body Mass Index; TNF, Tumor Necrosis Factor; IL, Interleukin; COPD, Chronic Obstructive Pulmonary Disease. Model 1: −2 Log likelihood = 5137.3, Chi-Square = 88.6, p < 0.001. Model 2: −2 Log likelihood = 5117.8, Chi-Square = 106.4, p < 0.001. Model 3: −2 Log likelihood = 5111.9, Chi-Square = 112.9, p < 0.001.

^a^p < 0.05, ^b^ p < 0.01, ^c^ p < 0.001. Note: Univariate analysis was performed using the enter method, and multivariate analysis was performed using the forward stepwise method.

## Discussion

This research revealed that older age of disease onset and being biologic-naïve were associated with the increased probability of achieving PASI90 response. In contrast, anti-IL12/23 agent use negatively affected PASI90 relative to the anti-TNFs. The age of disease onset was found to be the strongest predictor of PASI90 response. Also, anti-IL23 and anti-IL17 classes, particularly anti-IL23 agents, seemed related to higher treatment efficacy.

In previous studies, the PASI score of psoriasis patients was found to be higher in early-onset psoriasis cases compared to late-onset psoriasis cases,^13^, ^14^, ^15^ and there are few studies with conflicting results evaluating the relationship between the age of disease onset and biological treatment response in psoriasis patients. In the study by Singh et al., treatment responses of early-onset psoriasis patients receiving etanercept and infliximab treatment were significantly higher than those of late-onset psoriasis patients. In contrast, no relationship was found between the age of disease onset and treatment responses in patients receiving ustekinumab and adalimumab treatment.^16^ On the contrary, Griffiths et al. proposed that the PASI75 at week 12 of etanercept treatment was found to be higher in early-onset psoriasis patients than in late-onset psoriasis patients.^17^ The authors consider that the finding that older age is associated with a more favorable treatment response in this study may be related to cases beginning at an early age being more commonly HLA-Cw6-positive and thus showing lower treatment responses.^18^, ^19^, ^20^

Most studies in the literature and the work suggested that the efficacy of biologic therapy is higher in biologic-naïve patients than in biologic-experienced patients.^21^, ^22^, ^23^, ^24^ This might be related to cross-resistance between biologic classes, probably due to common pathways being operative downstream of cytokine signaling. A recent meta-analysis of 40 studies examining the clinical characteristics associated with treatment response to biologics identified that older age, higher Body Mass Index (BMI), previous or current smoking, and prior exposure to biologic treatments negatively influenced the probability of achieving PASI90 response. Also, the authors emphasized the unclearness of how these characteristics affect treatment responses differently for different biological therapies. In the present study, smoking status and BMI were not found to be significantly associated with PASI90 response. On the other hand, diabetes mellitus and hypertension, though statistically near-significant, were negatively related to the PASI90 response in the univariate analysis. Therefore, it can be proposed that components of metabolic syndrome impact treatment response, and distinct results of the studies are attributable to environmental factors (e.g., dietary habits) and racial genetic variances.

In the present study, the likelihood of achieving a PASI90 response was found to be significantly higher in patients with palmoplantar involvement. Yet, the authors consider this result misleading because the palmoplantar area is relatively small to evaluate the PASI response correctly. Instead, the Palmoplantar Pustulosis Severity Index (PPSI) is used to assess the severity of palmoplantar involvement and treatment response,^25^ but the authors did not implement this scale in this study. On the other hand, extremity involvement was significantly associated with worse treatment response in univariate analysis, albeit not significant in multivariable analysis. Hence, this indicates the involvement area may have affected the assessment of treatment response.

Inhibition of cytokine pathways has been related to various adverse events, and tuberculosis merits particular attention. Tuberculosis is still an essential concern in underdeveloped or developing countries worldwide when treating psoriasis patients with biologics, and the risk is even higher with TNF inhibitors than with any other biologic class.^26^, ^27^ Therefore, anti-TNFs opted for specific groups of psoriasis patients, such as those having concomitant inflammatory bowel disease.^27^ Ustekinumab, an anti-IL12/23 agent, is one of the earliest biologics approved for treating psoriasis. It is mainly preferred due to its lower risk of tuberculosis reactivation compared to anti-TNF agents. However, studies demonstrated that IL12 inhibition is unnecessary for psoriasis treatment,^28^ and IL12 inhibition may be associated with major cardiovascular events.^29^ On the other hand, the authors and others found that ustekinumab was associated with lower rates of PASI90 response.^30^

In the present study, the proportion of patients achieving PASI90 and PASI100 with biologic therapy was consistent with that reported in the literature.^21^, ^22^, ^23^, ^30^ The authors also observed the maximum treatment response after the 48th week of biologic therapy. Other studies were in line with the research and they reported that the maximum therapeutic efficacy of IL23 inhibitors, as well as anti-IL17 and anti-IL12/23 agents, was reached after the 48th week of treatment.^31^, ^32^, ^33^, ^34^, ^35^, ^36^ Notably, the distribution of anti-IL23 (guselkumab and risankizumab) and anti-IL17 agents (secukinumab and ixekizumab) was balanced in the present study, eliminating the bias risk within these classes. However, the frequencies of anti-TNF agents were fairly distinct, potentially leading to heterogeneity in the evaluation of treatment responses to this group.

The efficacy of anti-IL23 agents was significantly higher compared to the anti-TNFs and ustekinumab in the present work. Likewise, randomized controlled trials and real-world studies showed that the PASI90 response of anti-IL23 agents was higher than that of both anti-TNF and anti-IL12/23 classes.^37^, ^38^, ^39^, ^40^, ^41^, ^42^ The overall efficacy of anti-IL23 and anti-IL17 agents in patients with psoriasis in this study was found to be similar, and the literature presents varying results regarding the comparison of the efficacy and safety of these agents. In a meta-analysis, while IL17 inhibitors were found to have higher efficacy than IL23 inhibitors, IL23 inhibitors were considered safer regarding their side effect profiles.^43^ Although some studies have found the 12‒16th week PASI responses of IL23 inhibitors to be similar to those of IL17 inhibitors, the long-term efficacy of IL23 inhibitors has been reported to be higher.^44^, ^45^, ^46^ In a randomized, double-blind study, though the overall efficacy of IL17 inhibitors and IL23 inhibitors was found to be similar, IL17 inhibitors achieved much faster clearance of skin lesions.^47^ Also, a meta-analysis showed that risankizumab, followed by ixekizumab, provides the highest rates of PASI90 and PASI100 responses compared to other biologics.^30^ The present work was consistent with these findings, and IL17 inhibitors demonstrated a quicker PASI90 response than other classes. Furthermore, anti-IL23 agents had the highest treatment effectiveness, but no significant difference was observed in the overall efficacy of IL23 and IL17 inhibitors. However, the proportion of biologic-naïve patients was lowest in the anti-IL23 group in this study. Considering that biologic-naïveness significantly and positively affects the PASI90 response, the efficacy of the anti-IL23 agents can be expected to be higher if the number of biologic-naïve patients is evenly distributed across the biologic classes.

Several studies found that the IL-17 inhibitors provide higher rates of PASI90 compared to anti-TNF agents.^48^, ^49^, ^50^ Likewise, IL17 inhibitors exhibited higher performance for reaching PASI90 response than anti-TNFs in this study, but the result remained statistically near-significant. The lower proportion of biologic-naïve patients in the IL17 inhibitor group compared to the anti-TNF group might explain why statistical significance could not be achieved in the present study. Also, studies showed that the rates of PASI90 responses with IL17 inhibitors were higher than those of ustekinumab, which is compatible with this research.^51^, ^52^, ^53^, ^54^, ^55^

The most important limitation of this study is the retrospective data collection. The age groups of the study participants are heterogeneous. Also, the distribution of different anti-TNF agents and biologic-naïve patients is unbalanced, which might have confounded the results. On the other hand, the inclusion of a large number of patients from a single center and the evaluation of treatment responses by a few researchers are strengths of this study.

## Conclusion

In conclusion, age of disease onset, biologic-naïve, and ustekinumab use were identified as the independent predictors of PASI90 response, and age of disease onset was the most important. Anti-IL23 and anti-IL17 agents, especially IL23 inhibitors, exhibited superior therapeutic efficacy compared to other classes. This study underlines the necessity of head-to-head comparing the effectiveness of different biological agents with well-designed prospective trials.

## Financial support

None declared.

## Author's contribution

Burhan Engin: Wrote the manuscript, treated and followed the patients, interpreted the results, conceived the study, and approved the final version of the manuscript.

Yusuf Demir: Performed data collection, and approved the final version of the manuscript.

Sera Nur Yucesoy: Wrote the manuscript, and approved the final version of the manuscript.

Tumay Ak: Performed the statistical analysis, interpreted the results, conceived the study, edited the manuscript, and approved the final version of the manuscript.

## Conflicts of interest

None declared.
